# Wagner's Classification as a Tool for Treating Diabetic Foot Ulcers: Our Observations at a Suburban Teaching Hospital

**DOI:** 10.7759/cureus.21501

**Published:** 2022-01-22

**Authors:** Priti Shah, Ramteja Inturi, Dinesh Anne, Digvijay Jadhav, Varsha Viswambharan, Reina Khadilkar, Anuradha Dnyanmote, Shivangi Shahi

**Affiliations:** 1 Surgery, Dr DY Patil Vidyapeeth, Pune, IND

**Keywords:** diabetes, amputations, peripheral vascular disease, neuropathy, ulcer, diabetic foot

## Abstract

Objectives

The study aims to scale patients with diabetic foot ulcers according to Wagner’s classification, measure the various risk factors, study various outcomes and improve the treatment measures.

Methodology

The article presents materials on a prospective observational study of 50 diabetic foot patients with different presentations who underwent stage-specific intervention.

Results

Poor glycemic control, lifestyle factors, and smoking showed increased risks for foot ulcer complications. Diabetic neuropathy and vasculopathy have been significant outcome predictors. As a result, advanced Wagner’s grades showed increased amputation risks and multimodal management.

Conclusions

Stratification of diabetic foot patients and appropriate management based on their Wagner’s grade helps reduce amputation rates and mortality. In addition, multimodal management and exceptional attention to diabetes and lifestyle control improve long-term outcomes.

## Introduction

Diabetic foot infections are a significant cause of non-traumatic amputations and are preventable. Diabetes has become a substantial threat due to variations in demography, culture, and aging factors. It possesses a substantial economic burden and is a primary causative factor in cardiovascular diseases, amputations, blindness, and renal disorders. World Health Organization (WHO) reported over 20 million neuropathies, approximately six million amputations performed, and five million retinopathies associated with Diabetes [1].

Foot ulcer and gangrene are among the most severe complications of diabetes, with deaths almost the same as deaths due to cancers [2]. Additionally, individuals who have diabetes also have delayed wound healing. This leads to complications of diabetic foot with varying degrees of presentation.

Although many classifications are available for assessment, Wagner’s classification is a simple and widely accepted tool for evaluating diabetic foot lesions and effectively treating them [3].

The study aims to evaluate the different lesions of the diabetic foot according to Wagner’s classification, study the bacteriological profile of septic diabetic foot, provide different management options used for diabetic foot, compare outcomes, and identify measures to decrease morbidity.

## Materials and methods

Study design

We conducted a hospital-based prospective observational study at our tertiary care hospital after obtaining approval from the Institutional Ethics Sub-Committee (IESC) of Dr. D. Y. Patil Medical College, Hospital & Research Centre, Pune, IND. The approval number assigned to our study is IESC/PGS/2019/80.

Inclusion criteria

Patients of both genders and all age groups diagnosed with diabetic foot ulcers who had undergone detailed examination, routine investigations, and intervention were included in our study.

Exclusion criteria

Patients with ulcers pertaining to trauma and other neurological pathologies have been excluded from the study.

Data collection

We conducted a prospective study in our institute from September 2019 to August 2021, following approval from the hospital's ethical committee. We explained the subjects included, the course, and the study's aim. In addition, we described the patient information sheet and have taken the written consent form before actual participation.

We selected 50 patients from September 2019 to August 2021 from the outpatient clinics and wards and thoroughly explained the study process. We collected data by clinical history-taking, detailed examination, blood investigations, an X-ray of the foot, and all the details were noted down as per the proforma. Patients suspected of neuropathy on clinical examination underwent nerve conduction studies. Patients suspected of arterial involvement underwent a Doppler ultrasound. The patients were evaluated and managed according to Wagner's grade with surgical options ranging from debridement, incision, and drainage to below-knee amputation. See Table 1.

**Table 1:** Wagner's classification of diabetic foot ulcers

Wagner’s Classification		
Grade 0	Skin intact but bony deformities lead to "foot at risk"	
Grade 1	Superficial ulcer	
Grade 2	Deeper, full thickness extension	
Grade 3	Deep abscess formation or osteomyelitis	
Grade 4	Partial Gangrene of forefoot	
Grade 5	Extensive Gangrene	

We followed up for a minimum of three months. Any unwilling patient was allowed to leave the study anytime.

Data analysis

We collected the data in Microsoft Excel and analyzed using Statistical Package for the Social Sciences (SPSS) version 16 (SPSS Inc., Chicago, IL). Quantitative data were presented as mean ± standard deviation (SD), and qualitative data were presented as frequency. The unpaired T-test was used to compare normally distributed continuous variables between groups and consider a p-value less than 0.05 as significant.

Discussion and interpretation

After considering the study type, materials, methods, and results from appropriate, relevant studies, the study results were discussed in detail, and conclusions were drawn accordingly.

Ethics

Approval from the ethical committee of the college was taken beforehand. The patient's consent was taken only after explaining the study and the course. Patients were assured their reports would be kept confidential.

## Results

**Figure 1:**
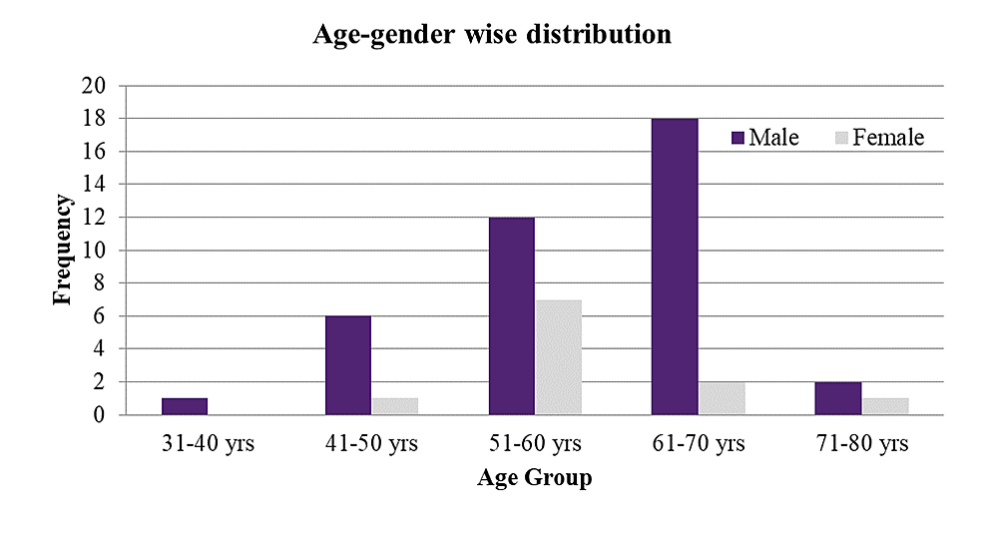
Bar diagram showing the age and gender-wise distribution of the study group

The mean age was 58.96 years (standard deviation - 8.352 years), with the oldest being 73 years and the youngest 37 years. Thirty-nine subjects (78%) were male while 11 (22%) were female. Twenty (40%) samples were from the age group of 61-70 years, followed by 19 (38%) subjects in the 51-60 years age group. See Figure 1.

**Figure 2:**
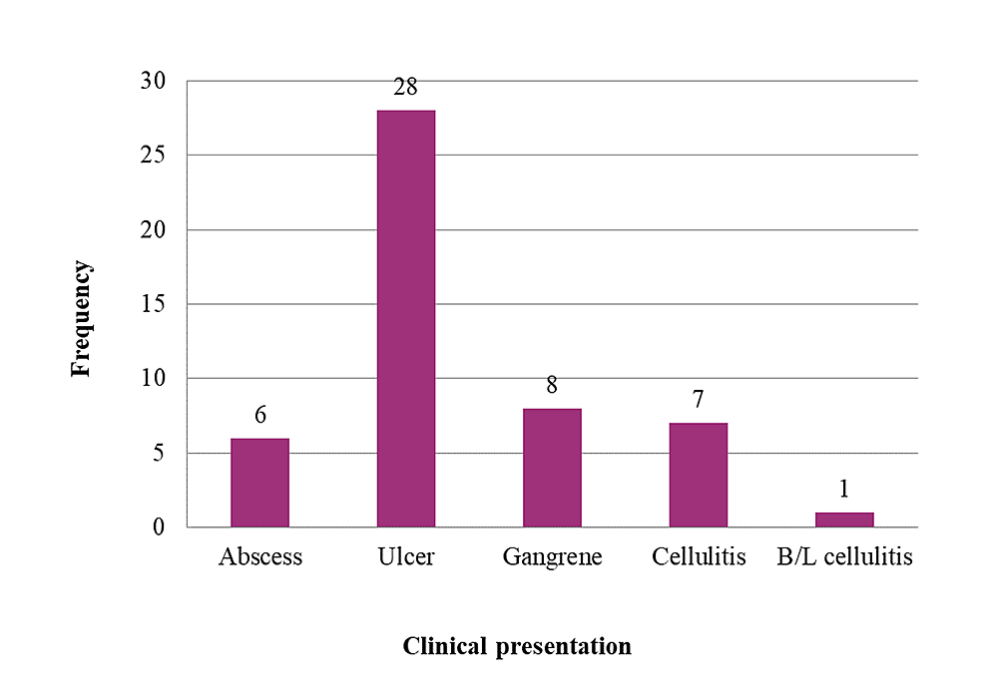
Bar diagram showing clinical presentation among the study sample

Foot ulcer was the most common presentation among study subjects, and it was present in 28 (56%) subjects, followed by gangrene with or without an ulcer (eight subjects), cellulitis (seven subjects), and abscess (six subjects). Bilateral (B/L) cellulitis was present in one subject. See Figure 2.

**Figure 3:**
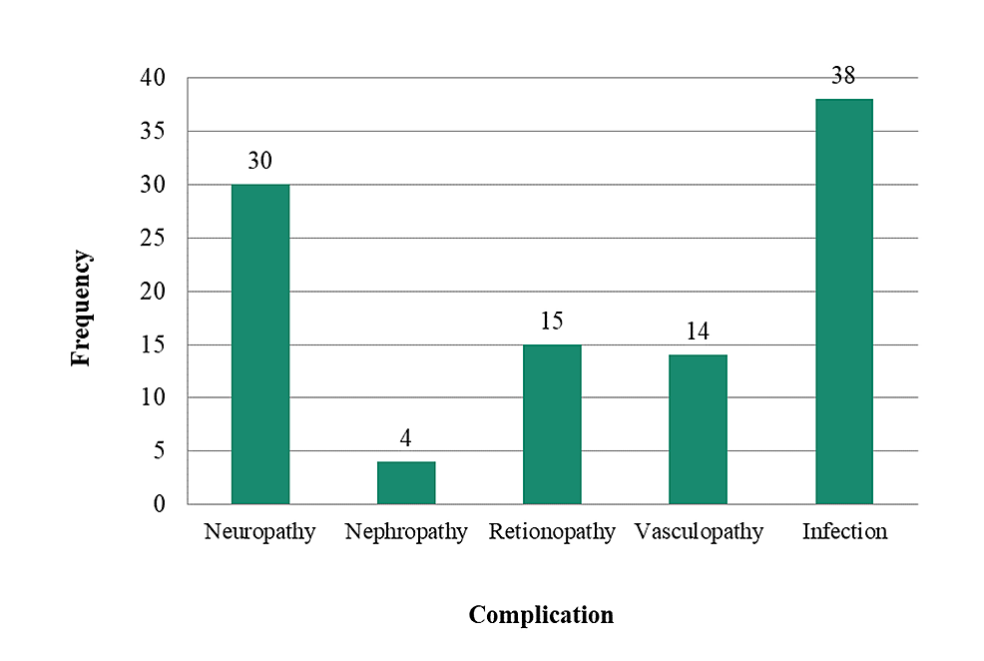
Bar diagram showing complications due to diabetes among the study sample

The presence of infection over the foot was the most common complication of diabetes among study subjects with 38 (76%) subjects, followed by neuropathy (30 subjects) and retinopathy (15 subjects). Peripheral vasculopathy was seen (14 subjects), and nephropathy (four subjects) was also present in some subjects. Some subjects had more than one complication. Diabetic nephropathy and retinopathy, although have no role in foot ulcer pathology, might indicate uncontrolled diabetes in the patient and increased amputation risks. See Figure 3.

**Figure 4:**
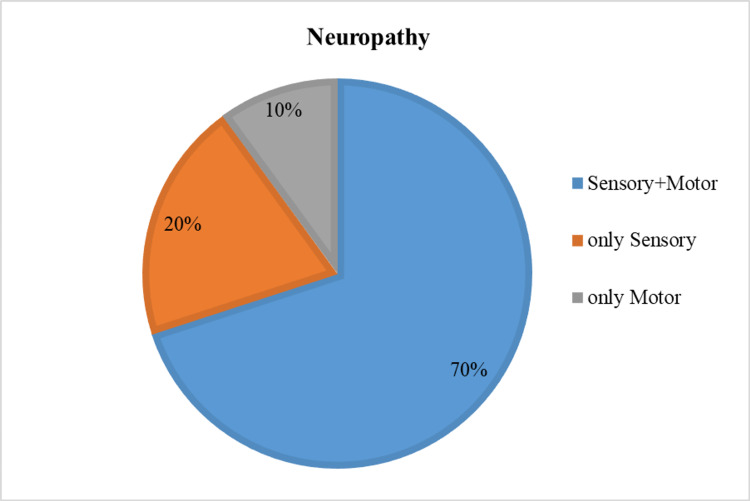
Pie chart showing neuropathy according to nerve conduction studies

Following clinical suspicion of neuropathy by examination with a Semmes Weinstein Monofilament test, it was confirmed with nerve conduction studies in 30 subjects. Twenty-one (70%) subjects showed both sensory and motor neuropathy while six subjects showed only sensory neuropathy and only motor neuropathy was noted in three subjects. See Figure 4.

**Figure 5:**
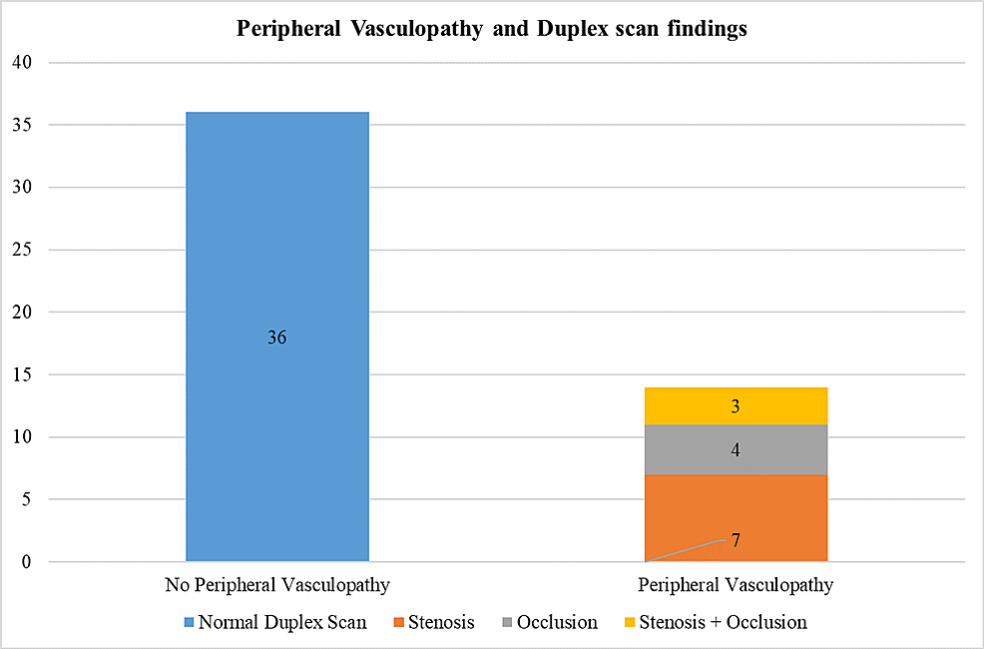
Bar diagram showing peripheral arterial involvement

Peripheral vasculopathy was assessed by clinical examination coupled with a duplex ultrasound scan. Fourteen subjects were identified to have peripheral vascular involvement (28%), and 36 subjects (72%) showed a normal scan. Out of the 14 subjects, stenosis was seen in seven patients (50%), with four subjects showing complete occlusion of the peripheral arteries and three subjects showing both stenosis and occlusion. See Figure 5.

**Figure 6:**
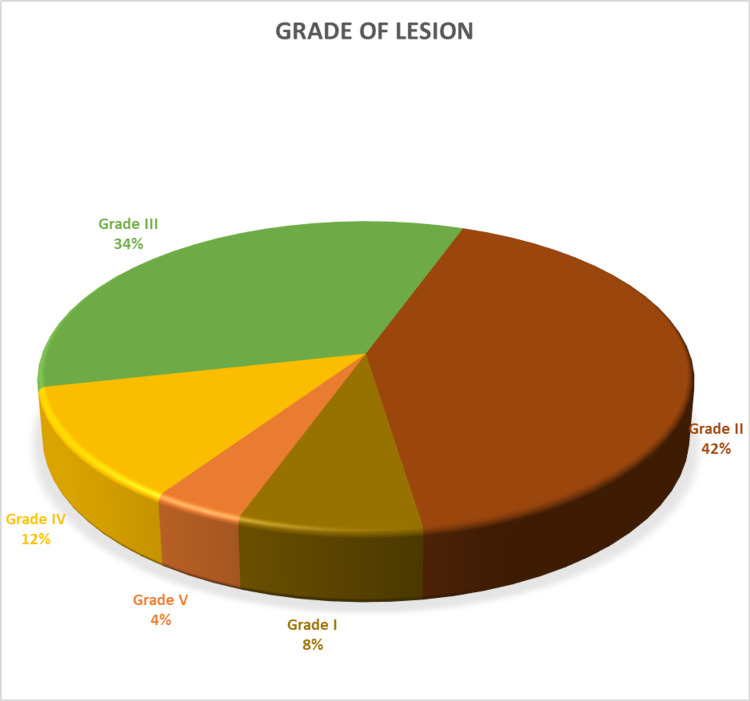
Pie chart showing grading according to Wagner’s classification

Twenty-one (42%) subjects had Wagner's grade II lesion followed by 17 (34%) with a grade III lesion and six (12%) with a grade IV lesion. Two (4%) subjects had extensive gangrene involving the foot due to diabetes. See Figure 6.

**Figure 7:**
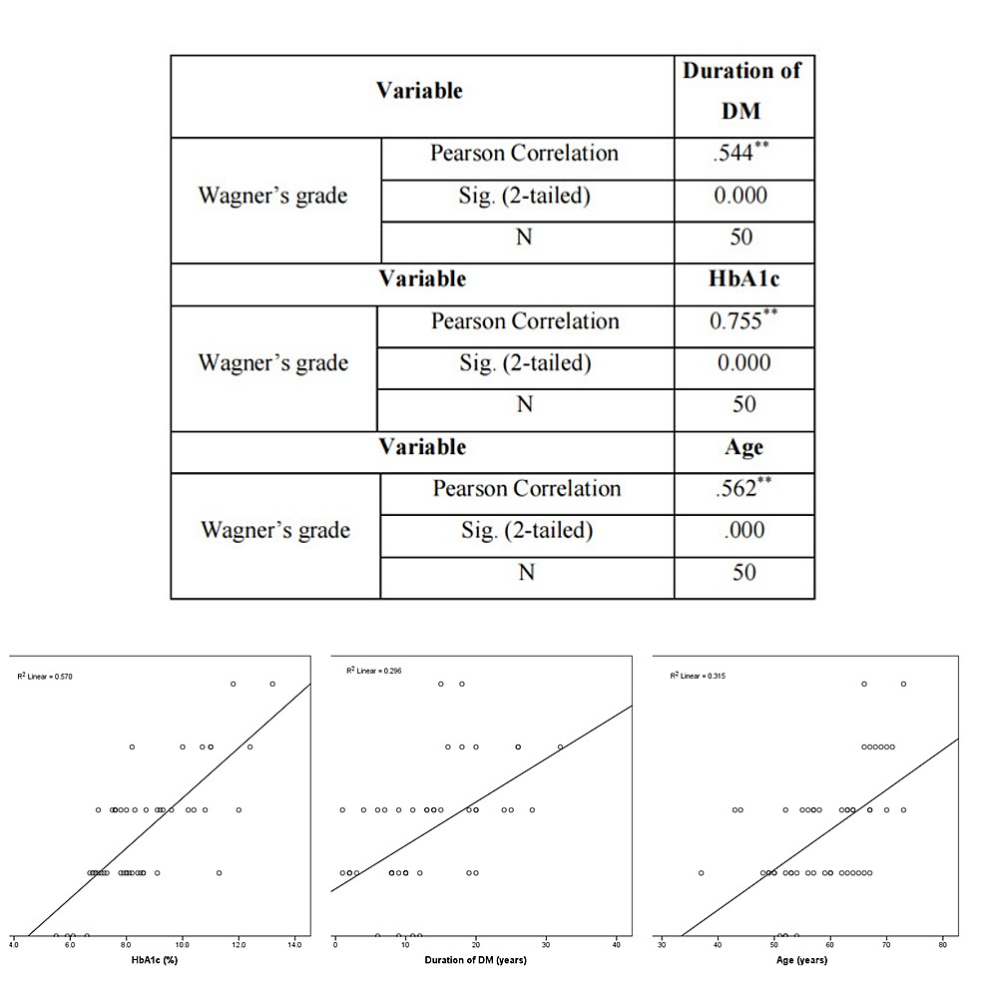
Graphs and table showing the correlation between Wagner's grading and age, duration of diabetes, and HbA1c levels

There was a significant positive correlation between Wagner's grading and advanced age, glycated hemoglobin (HbA1c), and duration of diabetes, suggesting an increased risk for amputation. See Figure 7.

Staphylococcus aureus was the most common organism infection in the study sample, followed by Pseudomonas and Escherichia coli (E.coli) in six subjects. In addition, Staphylococcus was frequently found in polymicrobial contamination. Finally, other organisms like Klebsiella pneumoniae, Bacteroides, and Streptococcus were also isolated in some subjects. See Table 2.

**Table 2:** Growth on blood culture

Growth on blood culture	Frequency
Bacteroides	2
Bacteroides + Pseudomonas	1
Escherichia coli	6
Enterococcus	1
Klebsiella + Acinetobacter	2
Klebsiella + Escherichia coli	3
Pseudomonas	6
Pseudomonas + Staphylococcus aureus	6
Staphylococcus aureus	9
Streptococcus agalactiae	2
Total	38

**Figure 8:**
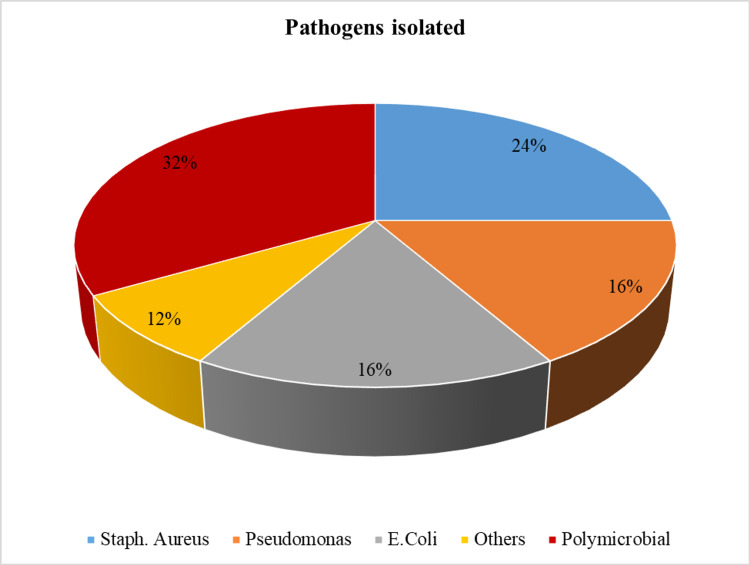
Pie chart showing different pathogens isolated from the wound

Twelve subjects were treated with oral hypoglycaemic drugs while the remaining 38 required insulin (Mixtard/Lantus/Actrapid), along with an antibiotic, selected according to the sensitivity to the isolated pathogen. See Figure 8.

Wound debridement (17) and amputation (14) was the common surgical treatment given to study subjects. Fasciotomy (7), daily dressing (6), and incision and drainage (5) were also part of the treatment for some study samples. See Table 3.

**Table 3:** Surgical management

Surgical management						
	Wagner’s grade					Total
Treatment	I	II	III	IV	V	
Amputation	-	-	6	6	2	14
Daily dressing	3	3	-	-	-	6
Debridement	1	15	1	0	-	17
Fasciotomy	-	1	6	-	-	7
Incision & drainage	-	1	4	-	-	5
Multiple debridements	-	-	-	-	1	1

**Figure 9:**
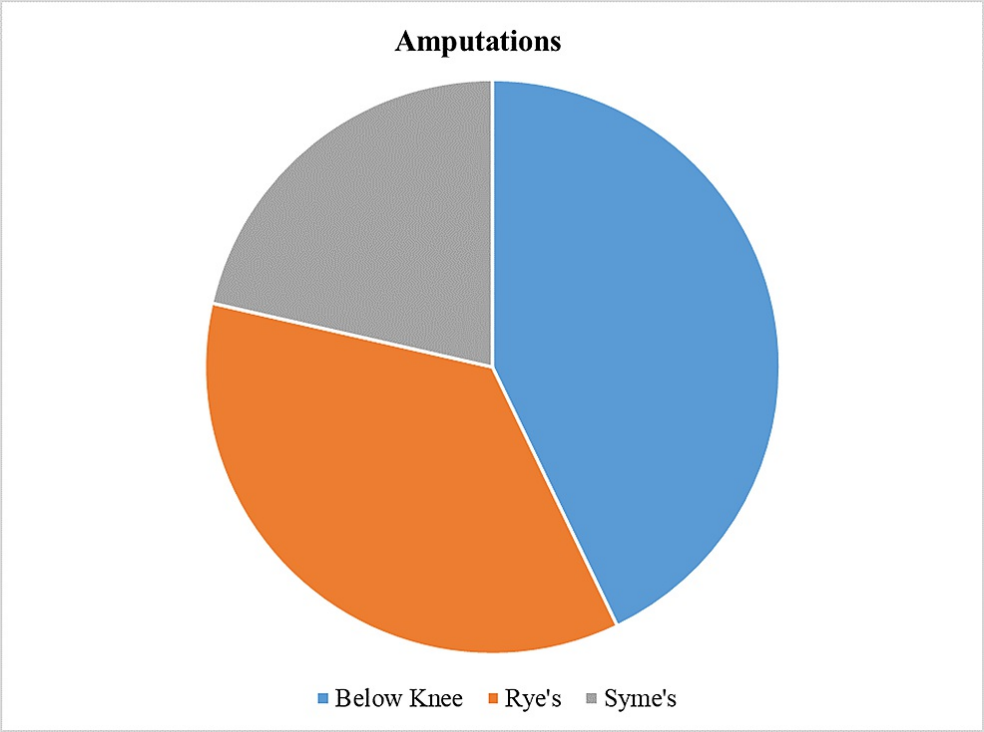
Amputations performed

Amputation was performed in 14 subjects with Wagner’s grade 3 ulcer or more, with six subjects undergoing below-knee amputation, five subjects undergoing Rye’s amputation, and three subjects subjected to Syme’s amputation. There were 16 subjects with Wagner’s grade 2 who underwent wound debridement. Some patients required multiple debridements and amputations. See Figure 9.

Following the primary management, 12 patients later underwent reconstruction, with nine patients undergoing split-thickness skin grafting (STSG) and three patients requiring some kind of flap.

## Discussion

India has the highest number of diabetics globally, accounting for one-sixth of all diabetic patients worldwide. Though diabetes affects multiple systems over time, foot ulcers and their complications have devastating morbidity and mortality [3].

Gouri et al. reported that 10.4% of all people with diabetes in rural parts of India presenting to a clinic have foot ulcers. On the whole, approximately 15% of the people with diabetes in India have been shown to develop a diabetic foot ulcer at some point in life, and these attribute to almost 85% of non-traumatic limb amputations [4].

In our study, comparative data of 50 patients were derived concerning diabetic foot ulcers. The mean age of 50 study samples was 58.96 years (standard deviation - 8.352 years). There were 39 (78%) males and 11 (22%) females in the study while 20 (40%) samples were from the 61-70 years age group followed by 19 (38%) subjects in 51-60 years age group, which shows that diabetes is more common in males and the older age groups.

This epidemiology was similar to Singh A et al., which showed most of the patients between the age of 45-64, with a male to female ratio of 2.28:1 [5]. In the study by Yerat al., male preponderance was seen with approximately a 1.5:1 ratio, showing almost 60.5% male subjects and 39.5% females with a mean age around 54.9 years [6].

Our study showed that Wagner’s grade 2 lesion, i.e., ulcer in the foot, was the most common lesion on presentation in 42% of cases (n=21), whereas a grade 3 lesion was seen in 34% of patients (n=17) and grade 4 lesions in 12% of patients (n=6). Akhter et al. reported the prevalence of diabetic foot at 11%, with 84% of subjects being men [7]. Again, the grade 2 lesion was the most common (34.5%).

Most patients of advanced grade (3-5) showed poorly managed diabetes, with peripheral neuropathy and peripheral vasculopathy being significant predictors of diabetic foot. In our study, diabetic neuropathy and vasculopathy were seen more in patients with increased HbA1c, advancing age, increased random blood sugar, and increased duration of diabetes mellitus and showed increased amputation risk in such patients. Farooque et al. reported that 26.13% of patients showed foot ulcerations, with nephropathy, peripheral neuropathy, ophthalmopathy, associated hypertension seen in 21.59%, 14.77%, 10.22%, and 15.9%, respectively [8].

Our study attempted to find the correlation of multiple risk factors of diabetes mellitus with grades of the disease and increased severity. We tried to derive the correlation using Pearson’s coefficient. Our study found a strong positive correlation between increasing grades of Wagner’s with advancing age. There was also a positive correlation between increased random blood sugar and increased duration of diabetes mellitus. Also, a higher HbA1C had a positive correlation with higher grades of Wagner’s classification suggesting uncontrolled diabetes presented severe types of diabetic foot ulcers, including gangrene.

Waghmare et al. also reported in their study of 47 patients that seven patients (14.9%) had blood sugar < 100 mg/dl [9]. Thirty-two patients (68.1%) had blood sugar between 101 and 200 mg/dl. Casadei et al. reported that increased HbA1c levels showed increased severity of neuropathy and poor glycemic control (HbA1c level >6.5), suggesting that an increased level of HbA1c can cause peripheral neuropathy [10]. Such patients with raised HbA1c are at high risk for foot ulceration. Subjects with lower HbA1c showed quicker healing time for foot ulcers. Markuson et al. reported HbA1c as a tool for determining healing time in diabetic foot ulcers [11].

Peripheral sensory neuropathy in the face of unperceived trauma is the primary factor leading to diabetic foot ulcerations [12]. Approximately 45% to 60% of all diabetic ulcerations are purely neuropathic while up to 45% have neuropathic and ischemic components. According to a prospective multicenter study, sensory neuropathy was the most frequent component in the causal sequence to ulceration in diabetic patients [12]. Also, foot deformities resulting from neuropathy, abnormal biomechanics, congenital disorders, or prior surgical intervention may result in high focal foot pressures and increased risk of ulceration [13].

Peripheral arterial disease (PAD) rarely leads to foot ulcerations directly. However, arterial insufficiency will result in prolonged healing once ulceration develops, imparting an elevated risk of amputation [14]. Additionally, attempts to resolve any infection will be impaired due to lack of oxygenation and difficulty delivering antibiotics to the infection site. Therefore, early recognition and aggressive treatment of lower extremity ischemia are vital to lowering limb salvage [15].

Akhter et al. also found that most patients lie between 40 years and 80 years of age [7]. More than 60% of patients showed uncontrolled hyperglycemia, with the study also showing male preponderance, and grade 2 lesions (34.5%) were the most common, consistent with our study.

Microbiological assessment of patients presenting with a diabetic foot was done, and it was found that Staphylococcus aureus was the most common organism, followed by E. coli, pseudomonas aeruginosa, and Klebsiella. Mixed culture was also isolated. Antibiotic sensitivity was also done. However, no fungal isolates were present in our study.

Yerat et al. reported that in their study, Gram-negative bacilli (78.98%) were more commonly isolated than Gram-positive cocci (21.01%) [6]. Proteus mirabilis was the most frequent organism isolated (26.08%). Bacteroides and Peptococcus were the commonest anaerobes obtained.

Most of our diabetic patients were managed well on oral hypoglycemic agents along with insulin (n=38), but a small group of patients was treated only on oral hypoglycemic agents. As evident from the above results, there is a strong correlation between the severity of foot ulcers and the extent of hyperglycemia. Hence, reasonable glycemic control is imperative for patients with advanced grades of ulcers.

The treatment and management of diabetic foot ulcers ultimately depend on the staging as per Wagner’s classification. As most of our patients were grade 2, ulcer debridement (n=17) was the most common procedure performed. Below-knee amputation (n=14) was performed in patients presenting with grades 3 to 5. Daily dressing was performed in a few patients (n=6), and fasciotomy was needed in seven patients. Amputation is needed more often in patients with pre-existing peripheral arterial disease.

A study by Rajyalaksmi et al. showed debridement as the choice of surgery in 38% of subjects, followed by disarticulation of toes for gangrene in 20% [16]. Seven percent of subjects needed Incision and drainage, and 5% of subjects required a major amputation. Chronic kidney disease patients with diabetic foot disease have a 10 times higher risk of leg amputation than the average population [17].

Gupta et al., in 100 patients with diabetic foot ulcers, found that most of their patients were grade 4 on presentation [18]. Glycemic control with anti-diabetics, the addition of appropriate antibiotics, and limb care were followed in 25 patients, and the rest of the 75 subjects required surgical management. Debridement or incision and drainage of the abscess was required in 41% of subjects and 35% of subjects undergoing an amputation, most commonly a below-knee amputation. Also, 5% of subjects required multiple revision surgeries.

Other adjunctive therapy like vacuum-assisted closure (VAC) dressing (negative pressure wound therapy), biological debridement by maggots, and other options are also available, with preliminary results encouraging. For example, the usage of hyperbaric oxygen coupled with laser therapy in chronic ulcers caused by diabetes showed almost 81% curative rates [19]. Emphasis must be kept on timely evaluation, diabetes control, and prevention of ulcer formation by proper nail and leg care using diabetes-specific micro-rubber footwear and other techniques.

## Conclusions

Diabetic foot ulcers are a common complication of prolonged diabetes. A grade 2 lesion is the most frequently encountered Wagner’s grade in our study, followed by grade 3. In grades 4 and 5, though less frequently encountered, the patients had severe gangrene and required prompt surgical intervention. Debridement was the most common procedure performed, as most patients are Wagner's grade 2. Amputations were required in grade 3 and above subjects, with below-knee amputation the most frequently performed procedure.

Elevated HbA1c levels, pre-existing peripheral arterial disease, and neuropathy strongly correlate to advanced Wagner's grade and need for amputation. Therefore, as emphasized, prevention and education about foot care in both patients and treating physicians is of utmost importance along with a multidisciplinary approach to tackle diabetes mellitus.
